# Right Heart Recovery Post Lung Transplant With COVID-19-Related Acute Respiratory Distress Syndrome

**DOI:** 10.1155/2024/8483800

**Published:** 2024-11-16

**Authors:** Ambalavanan Arunachalam, Takahide Toyoda, Tanvi Nayak, Madeline Jankowski, Emily Jeong Cerier, Taisuke Kaihou, Anthony Joudi, Suror Mohsin, Anjana Yeldandi, Mrinalini Venkata Subramani, Catherine Myers, Rade Tomic, Ankit Bharat, Kameswari Maganti, Chitaru Kurihara

**Affiliations:** ^1^Department of Medicine, Division of Pulmonary and Critical Care Medicine, Northwestern University, Chicago, Illinois, USA; ^2^Department of General Thoracic Surgery, Chiba University Graduate School of Medicine, 1-8-1 Inohana, Chuo-ku 260-8670, Chiba, Japan; ^3^Department of Medicine, Division of Cardiology, Northwestern University, Chicago, Illinois, USA; ^4^Department of Surgery, Division of Thoracic Surgery, Northwestern University, Chicago, Illinois, USA; ^5^Department of Pathology, Northwestern University, Chicago, Illinois, USA; ^6^Department of Medicine, Division of Cardiology, RWJ Barnabas Health, New Brunswick, New Jersey, USA

**Keywords:** acute respiratory distress syndrome, lung transplant, primary graft dysfunction

## Abstract

**Background:** Right heart remodeling is noted in patients with severe COVID-19-associated acute respiratory distress syndrome (ARDS). There is limited information regarding right heart recovery following lung transplantation in this cohort.

**Methods:** Retrospective review of institutional transplant database from June 2020 to June 2022 was performed at Northwestern University in Chicago, Illinois. Demographic, laboratory, histopathologic, lung transplant outcomes, and pre- and postoperative echocardiographic data were recorded and analyzed.

**Results:** Of the 42 patients who underwent lung transplantation for COVID-19-related ARDS, 6 patients were excluded due to having either a single-lung transplant (*n* = 2), lobar transplant (*n* = 1), or dual-organ transplant (*n* = 1) or for missing postoperative TTE data (*n* = 2) and 36 were included in the study; there were no 90-day deaths, and the 1-year survival rate was 88.8%. Intraoperative hemodynamics data showed a mean pulmonary artery pressure of 49 ± 23 mm Hg. Preoperative echocardiography was evaluated at a median of 15.5 (10–34.3) (IQR) days preoperatively and 140 (108–201) days (IQR) postoperatively. RV size grade improved from an average of 1.7 ± 0.85 to 1.3 ± 0.6 (*p* < 0.05), while RV function improved from an average of 2.2 ± 1.2 to 1 ± 1 (*p* < 0.05). There was a reduction in RVSP from 46.5 ± 18 mmHg to 30.1 ± 7.8 mmHg (*p* < 0.05) and RV free wall strain showed improvement from −13.9 ± 6.1% to −18.5 ± 5.4% (*p* < 0.05).

**Conclusions:** The results showed that the RV size and systolic function demonstrate improvement with normalization in a relatively short period following lung transplantation for patients with COVID-19-associated ARDS.

## 1. Introduction

Severe COVID-19 infection is characterized by acute respiratory distress syndrome (ARDS) and respiratory failure of varying severity [1, 2]. In 2020, approximately 6%–10% of patients who had COVID-19 infection developed ARDS and required prolonged mechanical ventilation [3]. In addition, right ventricular (RV) dysfunction is a common complication in patients with ARDS occurring in 22%–50% of patients and is associated with a nearly 50% increase in mortality [4–6]. Severe COVID-19 infection can cause irreversible lung parenchymal damage that molecularly and structurally resembles end-stage idiopathic pulmonary fibrosis, and lung transplantation may be the only therapeutic option for COVID-19-associated ARDS [7]. Lung transplantation is an established treatment for several chronic end-stage lung diseases, including idiopathic pulmonary fibrosis, chronic obstructive pulmonary disease (COPD), cystic fibrosis, and pulmonary arterial hypertension (PAH). Before the COVID-19 pandemic, patients with ARDS were rarely considered for a lung transplant [8]. On May 9, 2021, a multinational consortium of transplant centers from the United States, Austria, Italy, and India proposed guidelines for considering lung transplantation in patients with COVID-19-associated ARDS [9]. Since then, several centers have performed lung transplants for COVID-19-associated ARDS, and we have reported a good survival rate in a carefully selected group of patients who received lung transplants for COVID-19-associated ARDS [10]. The pathophysiology of RV dysfunction in the context of ARDS is multifactorial. This includes interstitial pulmonary edema, hypoxemic and hypercapnic pulmonary vasoconstriction, thromboembolism, maladaptive vascular remodeling leading to increased pulmonary vascular resistance, and pulmonary hypertension (PH). In addition, therapeutic measures to provide positive pressure ventilation and using positive end-expiratory pressure (PEEP) for lung recruitment increase RV afterload. Patients with COVID-19 infection and RV dysfunction showed a threefold higher likelihood of all-cause mortality than patients with normal RV function [2]. Advanced PH affects the RV morphology, causing RV dilation, hypertrophy, tricuspid regurgitation, and septal deviation, with associated loss of right and left ventricular function [11]. Prior work has shown improved RV morphology, function, and pressure overload in patients with advanced RV remodeling associated with PAH after lung transplant [12]. As lung transplantation is a relatively new treatment for ARDS, recovery of right heart morphology and function postlung transplantation is unknown. In addition, the impact of pre-existing advanced RV remodeling and significant PH during immediate post-transplant recovery and graft function outcomes are unknown.

Compared to patients with RV dysfunction related to PAH, patients with COVID-19-associated ARDS have a relatively short course of illness leading to transplant. Thus morphological and functional recovery of the right heart system after surgery is expected to be immediate. Additionally, myocardial strain assessment is a sensitive marker of subclinical RV dysfunction and may be used to evaluate right heart function following lung transplantation [13]. This study used preoperative and postoperative echocardiographic data to examine structural and functional changes in the right heart and discuss the likelihood and timing of recovery of RV function. In addition, the feasibility of a lung transplant to COVID-19-associated ARDS with severe RV dysfunction was evaluated.

## 2. Methods

### 2.1. Study Design and Participants

This study was approved by The Northwestern University Institutional Review Board (STU00207250 and STU00213616). Because this is a retrospective study, the need for patient consent for data collection was waived by the Institutional Review Board. This retrospective case series was performed at Northwestern University Medical Center in Chicago, Illinois. All consecutive patients who underwent lung transplantation for COVID-19-associated ARDS were enrolled between June 2020 and June 2022. Patients with a pre- and postoperative transthoracic echocardiogram beyond 30 days were included for analysis. Multiorgan transplant and single-lung transplant recipients were excluded from this study.

### 2.2. Data Collection

Clinical and laboratory characteristics, treatment, and outcomes data were obtained from electronic medical records by the investigative team and reviewed by thoracic surgeons and transplant pulmonologists. The information recorded included demographic data, medical history, underlying comorbidities, laboratory findings, medical course, treatments administered, pulmonary hemodynamics, and transthoracic echocardiography. In addition, intraoperative procedures and postoperative complications were recorded.

### 2.3. Lung Transplantation Evaluation and Listing in COVID-19-Associated ARDS

ARDS was defined according to the Berlin definition [14]. All patients with COVID-19-associated ARDS were treated by a multidisciplinary team that included surgeons, infectious disease physicians, pulmonary and critical care physicians, and cardiologists over the entire duration of the illness before being considered for transplantation. The decision to initiate extracorporeal membrane oxygenation (ECMO) was made by a multidisciplinary ECMO team, which included transplant pulmonologists, thoracic surgeons, ECMO specialists, and intensivists. During the pandemic's peak, most patients were cannulated at various outside hospitals for refractory ARDS and transferred to our institution for inability to wean off ECMO and consideration for a lung transplant. Our practice was to apply veno-venous ECMO with RV support using a dual-stage right atrium to pulmonary artery cannula, the Protek-Duo (CardiacAssist, Inc.), due to severe pulmonary hypertension and RV dysfunction. We used this cannulation strategy because of the significant RV dysfunction in our patients with severe refractory ARDS and the additional potential for RV support, with flow directly to the pulmonary circulation. In addition, the single site cannulation strategy helped us to promote ambulation and rehabilitation in the intensive care unit [15].

A referral for lung transplantation was made when this multidisciplinary team concluded that there was no longitudinal evidence of lung recovery at least six weeks after the onset of COVID-19-associated ARDS, which is consistent with our previous study [7, 9, 10]. Lung transplant evaluation was performed according to the International Society for Heart and Lung Transplantation (ISHLT) guidelines, and all patients were discussed in a multidisciplinary meeting to proceed toward transplant listing [16]. The broad transplant criteria for patients with COVID-19-associated ARDS included people aged 70 years or younger, two consecutive lower respiratory fluid polymerase chain reaction tests negative for SARS-CoV-2, single-organ failure, no evidence of irrecoverable brain damage, and a body mass index less than or equal to 35. Before transplant listing, all patients with COVID-19-associated ARDS received pretransplant rehabilitation during hospitalization, achieving sufficient truncal strength to sit upright and move all four limbs against gravity.

### 2.4. Definition of Primary Graft Dysfunction

PGD was defined based on the ISHLT guideline [17], and graded by PaO_2_/FiO_2_ ratio as follows: Grade 1: PaO_2_/FiO_2_ ratio > 300; Grade 2: PaO_2_/FiO_2_ ratio is 200–300; Grade 3: PaO_2_/FiO_2_ ratio < 200. The use of ECMO for bilateral pulmonary edema on chest X-ray was classified as grade 3.

### 2.5. Echocardiographic Evaluation

Pre- and post-lung transplant echocardiographic (echo) data were reviewed by two independent, experienced level 3 trained cardiologists blinded to the study's results. All of the traditional left ventricular (LV) and RV measurements were performed according to the conventional American Society of Echocardiography (ASE) guidelines. Two-dimensional speckle-tracking echocardiography was performed on cart and remeasured using an offline, vendor-independent analysis program (TomTec Imaging Systems, Munich, Germany). Measurements of the interventricular septum, LV internal diameter at end-diastole, LV internal diameter at end-systole, and posterior wall were performed from the parasternal long axis images. Left atrial (LA) volume index was calculated from apical 4-chamber and 2-chamber views, respectively, at end-systole when the LA chamber size is at its greatest dimension, taking care to avoid foreshortening. The right atrial (RA) area and volume were measured from the end ventricular end-systole when the RA chamber was at its greatest dimension. From an RV focused view, RV size and function were assessed. The basal RV diameter and the end-systolic and end-diastolic RV areas to assess fractional area change (FAC) were measured. RV wall thickness was measured from a subcostal view zoomed on the RV mid-wall at end-diastole. RV size was measured in RV focused apical 4-chamber view. In most patients, RV size could not be assessed in parasternal long axis view, proximal outflow tract parasternal long and short axis views. RV size was empirically graded based on a four-point scale using the basal RV diameter as follows: 1 = normal (25–40 mm), 2 = mildly dilated (41–45 mm), 3 = moderately dilated (46–50 mm), 4 = severely dilated (> 51 mm), while RV function was also evaluated on a four-point scale using FAC as a 2D surrogate for RV ejection fraction (1 = normal (> 35%), 2 = mildly decreased (30%–34%), 3 = moderately decreased (25%–29%), 4 = severely decreased < 25%). Apart from qualitative grading, RV function was also evaluated by tricuspid annular plane systolic excursion (TAPSE), TDI-derived tricuspid lateral annular systolic velocity (RV S′) measured in the RV-focused view, and RV free wall strain is described as follows: The hemodynamic right-sided assessment included the measurement of RV-RA gradient in calculating RV systolic pressure (RVSP) using a modified Bernoulli equation from the peak tricuspid regurgitant velocity signal. Right atrial pressure (RAP) assessment was performed using the diameter and collapsibility of the inferior vena cava from the subcostal view. The RAP was added to RVSP to obtain pulmonary artery systolic pressure (PASP). Mitral inflow parameters were measured, including E and A waves and medial and lateral e′ velocity. The E/e′ ratio was calculated to assess LV filling pressures.

Left ventricular global longitudinal strain was measured after the acquisition of LV focused views optimizing endocardial borders in the apical two-chamber, three-chamber, and four-chamber views at a frame rate of 60–90 Hz, taking care to minimize heart rate variation of no more than 5 beats between each view. RV free wall longitudinal strain was measured in the RV-focused view.

### 2.6. Statistical Analysis

The sample size was equal to the number of patients treated during the study period, and no statistical sample size was calculated. Continuous variables were reported as mean ± standard deviation or median with an interquartile range of 1–3. The Kaplan–Meier method was used to estimate survival differences between non-COVID-19- and COVID-19-associated ARDS patients. Statistical analysis was performed using EZR (Saitama Medical Center, Jichi Medical University, Japan), a GUI in R (The R Foundation for Statistical Computing, Vienna, Austria) [18]. Wilcoxon signed-rank test was used to compare pre- and postoperative TTE parameters. Statistical significance was denoted for variables with *p* < 0.05.

## 3. Results

### 3.1. Study Population and Clinical Characteristics of Lung Transplant Recipient

Of 42 patients who underwent lung transplantation for COVID-19-associated ARDS, 36 were included for analysis. Six patients were excluded due to having either a single-lung transplant (*n* = 2), lobar transplant (*n* = 1), or dual-organ transplant (*n* = 1) or for missing postoperative TTE data (*n* = 2). Demographics and comorbidities of lung transplant recipients and donors can be found in Table 1. The median age of the recipient cohort was 51.1 ± 11.3 years old, 38.9% female, while the median age of the donor cohort was 30.8 ± 12.2 years old with 27.1% females (Table 1). Comorbidities of the transplant recipients included remote smoking history, hypertension, and diabetes. Three patients (8.3%) required dialysis during their critical illness; however, their renal function recovered and had a glomerular filtration rate > 50 mL/min/1.73 m^2^ at the time of transplant listing. Twenty-four patients (66%) were bridged to transplantation with VV-ECMO cannulation, and the average time on ECMO until lung transplantation was 80.5 days (IQR 41.5–127.0). The remaining 12 patients had severe post-COVID pulmonary fibrosis as a sequelae of COVID-19-associated ARDS and were either unable to be weaned off the ventilator (*n* = 4) or high-flow oxygen (*n* = 8) for a meaningful recovery. Pulmonary artery pressures recorded in the operating room at the time of lung transplant with an in-dwelling pulmonary artery catheter showed significant pulmonary hypertension with an average PASP of 64.1 ± 27.1 mmHg, pulmonary artery diastolic pressure of 41.4 ± 22.8 mmHg, and mean pulmonary artery pressure of 49.4 ± 23.2 mmHg.

### 3.2. Intraoperative and Postoperative Outcomes

All patients in the study cohort underwent bilateral lung transplantation on Veno-Arterial ECMO due to the severity of pulmonary hypertension. The average operative time was 8.3 (7.8–9.6) hours, and the average VA-ECMO time was 3.2 (2.7–3.8) hours. Patients required substantial amounts of transfusion products due to coagulopathy related to adherent fibrotic lungs at the time of explant. Nine of the patients (25%) had PGD grade 3. Weaning off the vent was 20 (13.5–17.5) days. In the immediate postoperative days, 16 patients (45%) were electively kept on VV-ECMO post-transplantation. Eighteen patients developed acute kidney injury (51%), out of which, 8 patients required dialysis (23%). Eight patients (25%) were taken back to the operating room primarily for chest wall bleeding complications. Overall post-transplant ICU stay was 20 (13.5–26.5) days, and post-transplant hospital stay was 29 (17.5–38) days.

### 3.3. Echocardiographic Parameters

**Figure 1:**
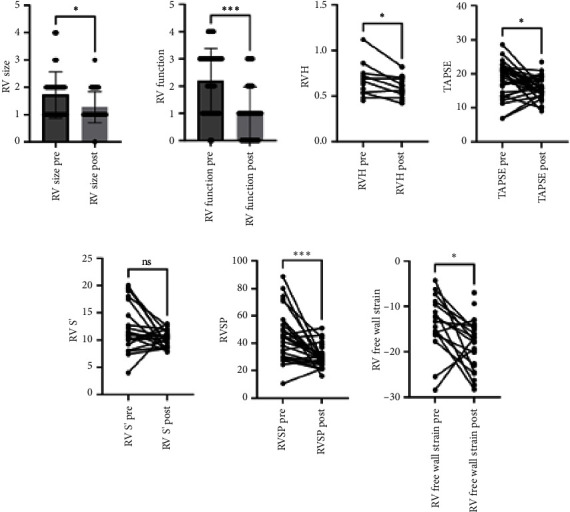
Pre- and post-transplant right ventricle echo parameters. RV G.L.S., right ventricular global longitudinal strain; RVH., right ventricular hypertrophy; RV S′, systolic tricuspid lateral annular velocity; RVSP, right ventricular systolic function; TAPSE, tricuspid annular plane systolic excursion.

Preoperative echo was evaluated at an average of 15.5 days before surgery (IQR 10.0–34.3), and the postoperative echo was assessed at an average of 140 days postoperatively (IQR 108–201). There were no significant differences in LV linear dimensions or LV ejection fraction (LVEF) following lung transplantation in this cohort. LV size at end diastole, LVEF, and left atrial volume index (LAVi) demonstrated an improvement when comparing pretransplant to post-transplant echos. There were significant changes in parameters such as mitral A wave velocity and lateral e′ velocity from 0.76 ± 0.21 m/s to 0.6 ± 0.16 m/s (*p* < 0.05), and from 9.4 ± 3.0 m/s to 11.4 ± 3.1 m/s (*p* < 0.05), respectively (Table 2). There was a significant improvement in RV size and systolic function, as seen in Table 2 and Figure 1. From the pretransplant to post-transplant echo, RV size grade improved from an average of 1.7 ± 0.85 to 1.3 ± 0.6 (*p* < 0.05), while RV function improved from an average of 2.2 ± 1.2 to 1 ± 1 (*p* < 0.05). In addition, RVSP was reduced from 46.3 ± 18 mmHg to 30.1 ± 7.8 mmHg (*p* < 0.05). RV free wall strain was obtained only for 16 patients due to difficulty with image acquisition, given artifacts noted from mechanical ventilation and RV support devices. RV free wall strain showed a statistically significant improvement between the pre- and post-transplant echo, with an initial value of −13.9 ± 6.1% and a follow-up value of −18.5 ± 5.4% (*p* < 0.05). Similarly, left ventricular global longitudinal strain demonstrated an improvement from −16.6 ± 4.0% to −17.6 ± 2.9% (*p* < 0.05).

#### 3.3.1. Explant Pathology

**Figure 2:**
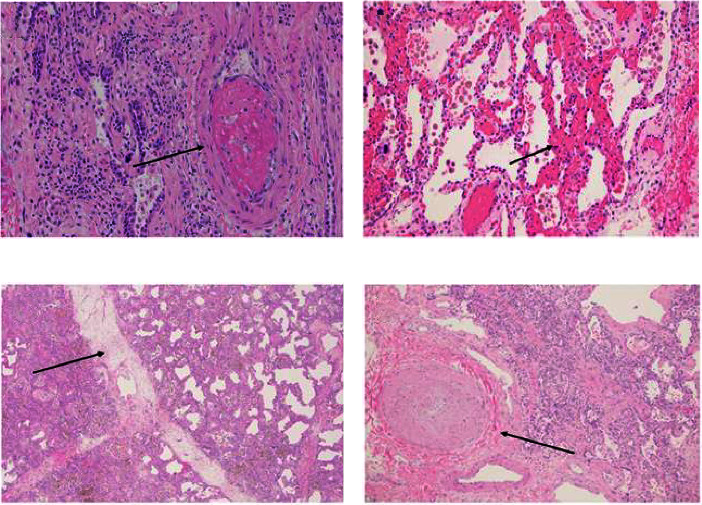
Explant pathology with pulmonary vascular remodeling. Arrows indicate (a) a nonocclusive microthrombus projecting into the vessel lumen, (b) expanded alveolar septum containing congested capillaries, (c) interlobular septal expansion, and (d) muscular hyperplasia and hypertrophy.

Explant histopathology assessment of patients with COVID-19-associated ARDS showed extensive alteration of the lung microstructure. There were several vascular changes consistent with severe pulmonary hypertension with resulting vascular remodeling. We selected a few explant histological slides to highlight the vascular remodeling changes including extensive microthrombi, capillary congestion and septal expansion, interlobular septal thickening, muscular hyperplasia, and hypertrophy (Figure 2). These findings overall signify the extent of vascular damage seen in COVID-19-associated ARDS.

### 3.4. Survival

**Figure 3:**
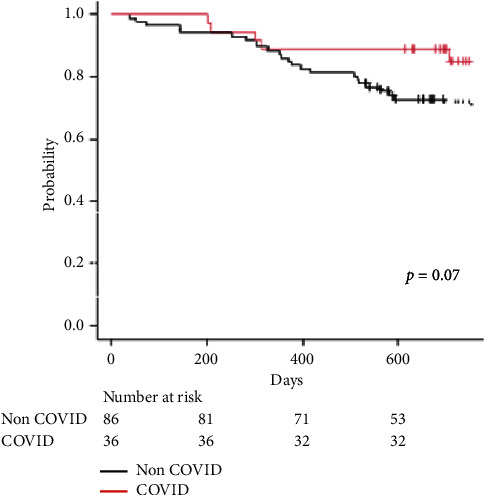
Kaplan–Meier survival curve: COVID-19-associated ARDS vs non-COVID patients.

The median follow-up period was 738 days (IQR 687–862) in the COVID-19-associated ARDS group vs 724 days (IQR 537–959) in the non-COVID group. As of July 1, 2023, 1-year survival rate of COVID-19-associated ARDS is 88.8% vs non-COVID 86.0% (*p*=0.07) per Figure 3.

## 4. Discussion

This single-center case series describes the clinical characteristics, intra- and postoperative outcomes, and post-transplant RV recovery of patients with severe PH and RV dysfunction associated with COVID-19-associated ARDS. This is the largest case series of patients with severe PH associated with COVID-19 ARDS. Before COVID-19, transplantation for refractory ARDS was a relatively rare indication for transplantation. Few retrospective case series have reported good outcomes for ARDS patients undergoing transplantation in the modern era [19]. We have demonstrated significant pulmonary hypertension in this cohort intraoperatively with PASP of 64 ± 27 mm Hg, PADP of 41 ± 23 mm Hg, and mean PAP of 49 ± 23 mm Hg. In addition, our histopathological specimens confirmed significant alveolar damage, microthrombi, and destruction, and signs of vascular remodeling which are typical changes seen in severe PH. ISHLT data reports that for patients receiving a lung transplant for the indication of idiopathic PAH between 1990 and 2014, the mortality risk in the first 3 months is higher for IPAH (23%) than for many other transplant indications, including chronic obstructive pulmonary disease (9%) or cystic fibrosis (9%) [20]. However, for transplant patients during the same era who survived to 1 year, conditional median survival was higher for idiopathic PAH (10.0 years) [20]. The higher mortality in the immediate postoperative period is due to the rapid physiological changes that occur upon implantation of donor lungs [21]. At the time of implantation of a healthy donor lung with normal pulmonary vascular resistance, cardiac output increases, which can result in immediate reperfusion edema and PGD. Also, due to the abnormal RV size, the intraventricular septum is shifted to the left, causing a reduction of LV dimension and stroke volume. We use various strategies to overcome these pathophysiological states in the immediate postoperative period and during liberation from mechanical ventilation. These include, reducing fluid load with diuresis, lung protective ventilation strategy, inotropic support for the RV and LV, inhaled nitric oxide, delayed extubation, and prolonged ECMO use in selective cases allowing the RV and LV time to adapt to the new pathophysiological conditions. We frequently monitored the RV and LV function with serial echocardiograms to assist with weaning supportive measures. Despite the high grade of PGD in this population, our data show that lung transplantation is technically feasible with good survival outcomes comparable to the non-COVID-19 group, and RV recovery occurs within 140 days after transplant. It is important to recognize that COVID-19-associated ARDS with RV dysfunction poses several other unique challenges toward safe transplantation; among them are pronounced comorbidities of these critically ill patients with malnutrition, neuromuscular deconditioning, severe pleural adhesions, and ventilator-associated bacterial pneumonia. These comorbidities significantly add to the complexity of the immediate post-transplant period and overall survival compared to patients with idiopathic PAH and secondary RV dysfunction. In our patient cohort, recovery of right heart size and function was evaluated by comparing defined echocardiographic parameters pretransplant to post-transplant. Following lung transplantation, we noted significant improvement in RV size and systolic function, RV mechanics, and RV hemodynamics. To our knowledge, this is the first study that reports RV remodeling post lung transplantation for ARDS. Prior studies have described improved right heart dilatation and function following lung transplant for patients with idiopathic PAH, as evaluated by similar echocardiographic parameters and cardiac MRI assessment. RV reverse remodeling occurs as early as 3 weeks post-transplant or can take up to a year [22–24]. In our study, TAPSE and RV lateral wall S′ did not show significant change following lung transplantation, highlighting the less sensitive nature of these measures to assess global RV function. We demonstrated that RV free wall strain showed significant improvement post-transplant, suggesting that RV reverse remodeling does occur post COVID19-associated ARDS. The time to normalization most likely parallels the extent of fibrosis noted within the RV myocardium. The significant improvement in lateral annular e′ velocity was considered secondary to improvement in LV function, paralleling improvement in RV function. This was not observed with medial annular e′ velocity, likely due to tethering of the septum due to changes in RV size, function, and overall mechanics. Mitral inflow A-wave decreased from pre to post-transplant, once again likely due to increased passive flow with improved LV function and a decreased active contribution from left atrial contraction. Other left heart parameters were also evaluated, including LVEF, LV GLS, and LAVi, which also showed improvement post-transplant. Left heart dysfunction following lung transplantation is a significant contributor to morbidity and mortality, and remains an important variable to monitor [25].

Since the principles of management of postoperative RV protective strategies and preserving graft function post-transplant are similar for non-COVID-19 ARDS, we believe these results and management strategies could be extrapolated widely to all refractory non-COVID19 ARDS patients who are being considered for lung transplant. However, the decision to proceed with lung transplant evaluation and surgery should be made by a multidisciplinary team which specializes in ARDS and lung transplant. Most patients with COVID-19-associated ARDS can demonstrate lung recovery with optimal ventilatory and extracorporeal support.

### 4.1. Limitations

This is a single-center, retrospective study, and the cohort sample size of 36 is small. There is a selection bias as only patients with interpretable echocardiograms pre- and post-transplant were included. Not all patients had optimal echocardiographic windows, which limited utility of all RV parameters, especially strain to assess for subclinical ventricular dysfunction. In addition, the post-transplant echo follow-up, on an average, was only 140 days. A longer duration of follow-up might demonstrate normalization of RV strain. The lack of validation cohort limits the outcome analysis. These patients were carefully selected and transplanted in a center experienced in managing these complex patients. Therefore, outcomes reported here might differ from national registries.

## 5. Conclusions

This study demonstrated significant RV dysfunction and pulmonary hypertension noted in patients who were carefully selected for lung transplantation from ARDS due to COVID-19. Lung transplantation reduces the hemodynamic burden on the right heart and allows RV and LV remodeling. Among the functional indices, significant improvements were more notable for RV size, function, and RV free wall GLS. This demonstrates that the structural and functional echocardiographic indices suggest reverse remodeling of RV with recovery in a relatively short period.

## Figures and Tables

**Table 1:** Characteristics of patients.

Variable	LTx patient for COVID-19-associated ARDS (*n* = 36)
Recipient factors	
Age, years	51.1 ± 11.3
Female	14 (38.9%)
BMI, kg/m^2^	26.6 ± 4.5
BSA, m^2^	1.9 ± 0.2
Smoking history	4 (11.1%)
Hypertension	15 (41.7%)
Diabetes	14 (38.9%)
Dialysis	3 (8.3%)
Pre-ECMO	23 (65%)
ECMO time to LTx (days)	80.5 (41.5–127)
LAS	82 ± 13.2
PASP (mmHg)	64.1 ± 27.1
PADP (mmHg)	41.4 ± 22.8
mPAP (mmHg)	49.4 ± 23.2
Donor age, years	30.8 ± 12.2
Donor sex, female (%)	13 (37.1%)
Intraoperative outcomes	
Operative time (hours)	8.3 (7.8–9.6)
Intraop blood transfusion; PRBC	7 (2.5–10)
Intraop blood transfusion; FFP	2 (0–5)
Intraop blood transfusion; platelet	2 (0–3)
Ischemic time (hours)	5.8 (5.3–6.1)
VA ECMO time (h)	3.2 (2.7–3.8)
Postoperative outcomes	
PGD grades 1 to 3	23 (65.7%)
PGD grade 3	9 (25.7%)
AKI	18 (51.4%)
Dialysis	8 (22.9%)
PE	1 (2.9%)
ICU stay (days)	20 (13.5–26.5)
Post-transplant ventilator (days)	4 (1–17.5)
Post plasmapheresis	12 (37.5%)
Hospital stay (days)	29 (17.5–38)
Post ECMO use	16 (45.7%)
Take back to OR	8 (25%)
Survival outcomes	
30-day survival rate	100.0%
90-day survival rate	100.0%
1-year survival rate	88.8%
Follow-up period (days)	738 (687–862)
LTx-pre echo (days)	15 (10–34)
Post echo-LTx (days)	140 (108–201)

*Note:* Continuous data are shown as means ± standard deviation (SD) and continuous data of days are shown as median (IQR).

Abbreviations: AKI, acute kidney injury; BMI, body mass index; BSA, body surface area; ECMO, extracorporeal membrane oxygenation; FFP, fresh frozen plasma; ICU, intensive care unit; LAS, lung allocation score; LTx, lung transplantation; mPAP, mean pulmonary artery pressure; PAD, pulmonary artery diastolic pressure; PAS, pulmonary artery systolic pressure; PE, pulmonary embolism; PGD, primary graft dysfunction; PRBC, packed red blood cell; OR, operating room.

**Table 2:** Echocardiographic parameters pre- and post-transplant.

Parameter	Prelung transplant (mean ± SD)	Postlung transplant (mean ± SD)	*p*-value
IVS (cm)	1.11 ± 0.39	1.05 ± 0.22	0.9448
LVIDD (cm)	4.4 ± 0.6	4.3 ± 0.6	0.5950
LVIDS (cm)	2.7 ± 0.5	3.6 ± 4.7	0.1212
PW (cm)	1.03 ± 0.15	1.06 ± 0.19	0.3350
LVEF-biplane (%)	64.8 ± 7.9	63.2 ± 6.5	0.1557
LAVI (mL/m^2^)	22.4 ± 8.8	28.1 ± 12.3	0.0297
LV GLS (%)	−16.6 ± 4.0	−17.6 ± 2.9	0.0312
Mitral E wave (m/s)	0.69 ± 0.18	0.76 ± 0.23	0.1752
Mitral A wave (m/s)	0.76 ± 0.21	0.6 ± 0.16	0.0016
Lateral e′ velocity (cm/s)	9.4 ± 3.0	11.4 ± 3.1	0.0122
E/e′	10.1 ± 3.5	10.9 ± 4.8	0.4562
Right ventricle parameters			
RV size (1–4)	1.7 ± 0.85	1.3 ± 0.6	0.0346
RV function-FAC (0–4)	2.2 ± 1.2	1 ± 1	0.0001
RVH	0.66 ± 0.18	0.60 ± 0.11	0.0312
TAPSE	18.9 ± 7.4	15.5 ± 5.4	0.0212
RV S′	12.1 ± 4.4	10.3 ± 1.5	0.1089
RVSP (mm Hg)	46.5 ± 18.6	30.1 ± 7.8	0.0001
RV free wall strain (%)	−13.9 ± 6.1	−18.5 ± 5.4	0.0155

*Note:* RV size (*1 = normal, 2 = mildly dilated, 3 = moderately dilated, 4 = severely dilated*); RV systolic function (*0 = low normal, 1 = normal, 2 = mildly decreased, 3 = moderately decreased, 4 = severely decreased*).

Abbreviations: GLS, global longitudinal strain; IVS, interventricular septum; LAVI, left atrial volume index; LVEF, left ventricular ejection fraction; LVIDD, left ventricular internal dimensions at end-diastole; LVIDS, left ventricular internal dimensions at end-systole; PW, posterior wall; RV, right ventricular; RVH, right ventricular hypertrophy; RVSP, right ventricular systolic function.

## Data Availability

Authors do not intend to share patient data with journal.

## Nomenclature

AKI: Acute kidney injury
ASE: American Society of Echocardiography
ARDS: Acute respiratory distress syndrome
BMI: Body mass index
CI: Confidence interval
COPD: Chronic obstructive pulmonary disease
ECMO: Extracorporeal membrane oxygenation
FAC: Fractional area change
GLS: Global longitudinal strain
IQR: Interquartile range
ISHLT: International Society for Heart and Lung Transplantation
LA: Left atrium
LV: Left ventricle
PAH: Pulmonary arterial hypertension
PASP: Pulmonary artery systolic pressure
PEEP: Positive end-expiratory pressure
PGD: Primary graft dysfunction
PH: Pulmonary hypertension
RA: Right atrium
RAP: Right atrial pressure
RV: Right ventricle
RSVP: Right ventricular systolic pressure.

## References

[1] Gupta S., Hayek S. S., Wang W. (2020). Factors Associated With Death in Critically Ill Patients With Coronavirus Disease 2019 in the US. *JAMA Internal Medicine*.

[2] Corica B., Marra A. M., Basili S. (2021). Prevalence of Right Ventricular Dysfunction and Impact on All-Cause Death in Hospitalized Patients With COVID-19: A Systematic Review and Meta-Analysis. *Scientific Reports*.

[3] Richardson S., Hirsch J. S., Narasimhan M. (2020). Presenting Characteristics, Comorbidities, and Outcomes Among 5700 Patients Hospitalized With COVID-19 in the New York City Area. *JAMA*.

[4] Zochios V., Parhar K., Tunnicliffe W., Roscoe A., Gao F. (2017). The Right Ventricle in ARDS. *Chest*.

[5] Sato R., Dugar S., Cheungpasitporn W. (2021). The Impact of Right Ventricular Injury on the Mortality in Patients With Acute Respiratory Distress Syndrome: A Systematic Review and Meta-Analysis. *Critical Care*.

[6] Mekontso Dessap A., Boissier F., Charron C. (2016). Acute Cor Pulmonale during Protective Ventilation for Acute Respiratory Distress Syndrome: Prevalence, Predictors, and Clinical Impact. *Intensive Care Medicine*.

[7] Bharat A., Querrey M., Markov N. S. (2020). Lung Transplantation for Patients with Severe COVID-19. *Science Translational Medicine*.

[8] van der Mark S. C., Hoek R. A. S., Hellemons M. E. (2020). Developments in Lung Transplantation over the Past Decade. *European Respiratory Review*.

[9] Bharat A., Machuca T. N., Querrey M. (2021). Early Outcomes after Lung Transplantation for Severe COVID-19: a Series of the First Consecutive Cases from Four Countries. *Lancet Respiratory Medicine*.

[10] Kurihara C., Manerikar A., Querrey M. (2022). Clinical Characteristics and Outcomes of Patients with COVID-19-Associated Acute Respiratory Distress Syndrome Who Underwent Lung Transplant. *JAMA*.

[11] Kasimir M. T., Seebacher G., Jaksch P. (2004). Reverse Cardiac Remodelling in Patients with Primary Pulmonary Hypertension after Isolated Lung Transplantation. *European Journal of Cardio-Thoracic Surgery*.

[12] Gorter T. M., Verschuuren E. A. M., van Veldhuisen D. J. (2017). Right Ventricular Recovery after Bilateral Lung Transplantation for Pulmonary Arterial Hypertension. *Interactive Cardiovascular and Thoracic Surgery*.

[13] Li Y., Li H., Zhu S. (2020). Prognostic Value of Right Ventricular Longitudinal Strain in Patients with COVID-19. *JACC: Cardiovascular Imaging*.

[14] Ranieri V. M., Rubenfeld G. D., Thompson B. T. (2012). Acute Respiratory Distress Syndrome: The Berlin Definition. *JAMA*.

[15] Cerier E., Manerikar A., Kandula V. (2023). Early Initiation of Physical and Occupational Therapy while on Extracorporeal Life Support Improves Patients’ Functional Activity. *Artificial Organs*.

[16] Leard L. E., Holm A. M., Valapour M. (2021). Consensus Document for the Selection of Lung Transplant Candidates: An Update from the International Society for Heart and Lung Transplantation. *The Journal of Heart and Lung Transplantation: The Official Publication of the International Society for Heart Transplantation*.

[17] Snell G. I., Yusen R. D., Weill D. (2017). Report of the ISHLT Working Group on Primary Lung Graft Dysfunction, part I: Definition and grading-A 2016 Consensus Group statement of the International Society for Heart and Lung Transplantation. *The Journal of Heart and Lung Transplantation: The Official Publication of the International Society for Heart Transplantation*.

[18] Kanda Y. (2013). Investigation of the Freely Available Easy-To-Use Software “EZR” for Medical Statistics. *Bone Marrow Transplantation*.

[19] Gottlieb J., Lepper P. M., Berastegui C. (2022). Lung Transplantation for Acute Respiratory Distress Syndrome: A Retrospective European Cohort Study. *European Respiratory Journal*.

[20] Yusen R. D., Edwards L. B., Kucheryavaya A. Y. (2015). The Registry of the International Society for Heart and Lung Transplantation: Thirty-Second Official Adult Lung and Heart-Lung Transplantation Report--2015; Focus Theme: Early Graft Failure. *The Journal of Heart and Lung Transplantation: The Official Publication of the International Society for Heart Transplantation*.

[21] Moser B., Jaksch P., Taghavi S. (2018). Lung Transplantation for Idiopathic Pulmonary Arterial Hypertension on Intraoperative and Postoperatively Prolonged Extracorporeal Membrane Oxygenation Provides Optimally Controlled Reperfusion and Excellent Outcome. *European Journal of Cardio-Thoracic Surgery*.

[22] Salman J., Ius F., Sommer W. (2017). Mid-term Results of Bilateral Lung Transplant With Postoperatively Extended Intraoperative Extracorporeal Membrane Oxygenation for Severe Pulmonary Hypertension. *European Journal of Cardio-Thoracic Surgery*.

[23] Frist W. H., Lorenz C. H., Walker E. S. (1995). MRI Complements Standard Assessment of Right Ventricular Function After Lung Transplantation. *The Annals of Thoracic Surgery*.

[24] Kusunose K., Tsutsui R. S., Bhatt K. (2014). Prognostic Value of RV Function Before and After Lung Transplantation. *JACC Cardiovascular Imaging*.

[25] Tudorache I., Sommer W., Kühn C. (2015). Lung Transplantation for Severe Pulmonary Hypertension--Awake Extracorporeal Membrane Oxygenation for Postoperative Left Ventricular Remodelling. *Transplantation*.

